# Host Protein BAG3 is a Negative Regulator of Lassa VLP Egress

**DOI:** 10.3390/diseases6030064

**Published:** 2018-07-13

**Authors:** Ziying Han, Michael P. Schwoerer, Philip Hicks, Jingjing Liang, Gordon Ruthel, Corbett T. Berry, Bruce D. Freedman, Cari A. Sagum, Mark T. Bedford, Sachdev S. Sidhu, Marius Sudol, Ronald N. Harty

**Affiliations:** 1 Department of Pathobiology, School of Veterinary Medicine, University of Pennsylvania, Philadelphia, PA 19104, USA; ziying@vet.upenn.edu (Z.H.); mschwo@sas.upenn.edu (M.P.S.); hicksph@vet.upenn.edu (P.H.); jiliang@vet.upenn.edu (J.L.); goruthel@vet.upenn.edu (G.R.); corbettb@vet.upenn.edu (C.T.B.); bruce@vet.upenn.edu (B.D.F.); 2 Department of Epigenetics & Molecular Carcinogenesis, M.D. Anderson Cancer Center, University of Texas, Smithville, TX 78957, USA; casagum@mdanderson.org (C.A.S.); mtbedford@mdanderson.org (M.T.B.); 3 Department of Molecular Genetics, University of Toronto, Toronto, ON M1C 1A4, Canada; sachdev.sidhu@utoronto.ca; 4 Department of Physiology, Institute for Molecular and Cell Biology (IMCB, AStar), National University of Singapore, Singapore 119077, Singapore; phsms@nus.edu.sg

**Keywords:** Lassa fever virus, VLPs, budding, BAG3, L-domain, virus-host interaction, Ebola, WW-domain, autophagy

## Abstract

Lassa fever virus (LFV) belongs to the *Arenaviridae* family and can cause acute hemorrhagic fever in humans. The LFV Z protein plays a central role in virion assembly and egress, such that independent expression of LFV Z leads to the production of virus-like particles (VLPs) that mimic egress of infectious virus. LFV Z contains both PTAP and PPPY L-domain motifs that are known to recruit host proteins that are important for mediating efficient virus egress and spread. The viral PPPY motif is known to interact with specific host WW-domain bearing proteins. Here we identified host WW-domain bearing protein BCL2 Associated Athanogene 3 (BAG3) as a LFV Z PPPY interactor using our proline-rich reading array of WW-domain containing mammalian proteins. BAG3 is a stress-induced molecular co-chaperone that functions to regulate cellular protein homeostasis and cell survival via Chaperone-Assisted Selective Autophagy (CASA). Similar to our previously published findings for the VP40 proteins of Ebola and Marburg viruses, our results using VLP budding assays, BAG3 knockout cells, and confocal microscopy indicate that BAG3 is a WW-domain interactor that negatively regulates egress of LFV Z VLPs, rather than promoting VLP release. Our results suggest that CASA and specifically BAG3 may represent a novel host defense mechanism, whereby BAG3 may dampen egress of several hemorrhagic fever viruses by interacting and interfering with the budding function of viral PPxY-containing matrix proteins.

## 1. Introduction

Viral hemorrhagic fever (VHF) is a severe syndrome that can be caused by a number of emerging RNA viruses including arenaviruses such as Lassa and filoviruses such as Ebola [1]. Indeed, both Lassa and Ebola viruses are zoonotic pathogens with the ability to cause severe VHF disease in humans with high case fatality rates [1]. Because of the severe pathogenic potential and limited treatment options for these hemorrhagic fever viruses, there remains an urgent need to understand the molecular mechanisms of these diseases, including a better understanding of the specific interactions between the virus and the host that may contribute to the disease process.

Both Lassa and Ebola encode matrix proteins that play key roles in virion assembly and egress from infected cells. Expression of either the Lassa Z or Ebola VP40 matrix protein alone is sufficient to generate the formation of Z or VP40 virus-like particles (VLPs), respectively, that are capable of budding from mammalian cells in a manner that closely mimics that of intact infectious virions [2,3,4,5,6,7,8,9]. Both Lassa Z and Ebola VP40 contain conserved amino acid motifs referred to as late (L) domains that have the core consensus motifs of either PTAP or PPxY (where x = any amino acid). The viral L-domains have been shown to facilitate budding of both VLPs and infectious virions by a mechanism that involves interactions between the L-domain motifs and specific host proteins [3,7,10,11,12,13,14,15,16,17,18,19,20,21,22,23,24,25,26,27,28,29,30,31,32]. For example, the viral PPxY motif interacts with specific host proteins containing one or more WW-domains, and the majority of host WW-domain interactors identified to date have been shown to enhance or facilitate VLP and/or virus egress. One notable exception is host protein BCL2 Associated Athanogene 3 (BAG3), a VP40 PPxY interactor and WW-domain containing co-chaperone protein that functions in maintaining cell survival and protein homeostasis via chaperone-assisted selective autophagy (CASA) [19,33]. Our recent findings demonstrated that BAG3 interacted with the PPxY motifs present in both Ebola and Marburg VP40 proteins, and negatively regulated egress of VP40 VLPs in a PPxY/WW-domain dependent manner [19]. Our data further suggested that BAG3 may dampen VLP egress by sequestering VP40 away from the site of budding at the plasma membrane, thus reducing the efficiency of filovirus VLP release [19]. Indeed, we found that in the presence of BAG3-WT, VP40 colocalized with microtubule-associated light chain 3 (LC3), a well-known marker for aggresomes [19].

Here, we have expanded on our efforts to elucidate the role of BAG3 as a negative regulator of virus budding by determining whether BAG3 can also negatively affect egress of PPxY-containing Lassa Z VLPs. Indeed, we identified BAG3 as a WW-domain interactor with the PPxY motif of Lassa Z by screening a GST array of 115 WW-domain bearing host proteins with either WT or PPxY mutant peptides of Lassa Z. We used peptide pulldown assays to confirm the PPxY/WW-domain dependent interaction between Lassa Z and host BAG3. We also demonstrated that exogenous expression of WT BAG3 inhibited egress of Lassa Z VLPs, whereas exogenous expression of a WW-domain deletion mutant of BAG3 had no effect on Lassa Z VLP egress. Conversely, we observed a modest yet consistent increase in Lassa Z VLP egress from HAP1 BAG3 knockout (KO) cells compared to that from parental HAP1 cells expressing normal levels of endogenous BAG3. In addition, we demonstrate that budding of eVP40 VLPs and infectious VSV recombinants expressing the eVP40 PPxY motif was also enhanced in BAG3 KO cells compared to that in BAG3 WT cells. Lastly, results from confocal microscopy studies suggest that release of Lassa Z VLPs was inhibited in cells co-expressing WT BAG3, whereas efficient release of VLPs was not inhibited in cells co-expressing a WW-deletion mutant of BAG3. Our data suggest that BAG3 functions to reduce efficient egress of Lassa Z VLPs in a manner mechanistically similar to that reported previously for Ebola and Marburg VLPs, and that CASA may function as a cellular defense mechanism against invading pathogens.

## 2. Materials and Methods

### 2.1. Cell Lines, Plasmids, and Reagents

HEK293T (American Type Culture Collection; ATCC, Manassas, VA, USA) cells were maintained in Dulbecco’s modified Eagle’s medium (DMEM) supplemented with 10% fetal calf serum (FCS), penicillin (100 U/mL)/streptomycin (100 µg/mL) at 37 °C in a humidified 5% CO_2_ incubator. Human wild-type (WT) HAP1 cells (kindly provided by K. Chandran, Albert Einstein College of Medicine, New York, NY, USA) and HAP1-BAG3 knockout (KO) cells (Horizon Discovery, Waterbeach, UK) were maintained in Iscove’s modified Dulbecco’s medium (IMDM) supplemented with 10% FCS and penicillin (100 U/mL)–streptomycin (100 μg/mL) at 37 °C in a humidified 5% CO_2_ incubator. The HAP1-BAG3 KO cells contain an 11 base pair deletion in the coding exon of BAG3. A truncated form of BAG3 lacking the N-terminal WW-domain is expressed in these cells from an internal ATG start codon. The pcDNA6 myc-His-BAG3-WT (1–575), pcDNA6 myc-His-BAG3-ΔN (62–575) and pcDNA6 myc-His-BAG3-ΔC (1–420) plasmids were kindly provided by K. Khalili (Temple University, Philadelphia, PA, USA) and were described previously [19]. The HA-tagged LFV-Z expression plasmid was kindly provided by S. Urata (Nagasaki, Japan) and was described previously [23]. Mouse anti-Myc antiserum (05-724) was purchased from EMD Millipore. Mouse anti-HA (H9658), rabbit anti-Myc (C3956), and mouse anti-β-actin (A1978) antisera were purchased from Sigma-Aldrich (ST. Louis, MO, USA).

### 2.2. PPxY-WW Domain Array

The WW- and SHS-domain array consisted of almost all known WW domains (115 domains) from mammalian and yeast proteins. We synthesized biotinylated peptides containing either the LFV Z WT PPxY motif (TAPPEIPPSQNPPPYSP-K-Biotin) or mutated PPxY motif (TAPPEIPPSQNAAPASP-K-Biotin) (Thermo Fisher, Waltham, MA, USA). The biotinylated peptides were fluorescently labeled and used to screen the recombinant domains that were arrayed in duplicate onto nitrocellulose-coated glass slides (OncyteAvid slides; Grace Bio-Labs, Bend, OR, USA). Fluorescence was detected using a GeneTAC LSIV scanner (Genomic Solutions, Ann Arbor, MI, USA).

### 2.3. Peptide Pull-Down Assay

Peptide pull-down assays were performed as described previously [18]. Briefly, streptavidin agarose beads (Millipore, Burlington, MA, USA) were pre-washed once with 1× mild buffer (50 mM Tris HCl pH 7.5, 150 mM NaCl, 0.1% NP-40, 5 mM EDTA, 5 mM EGTA, 15 mM MgCl_2_), and 15 μg of either biotinylated LFV-Z-WT or LFV-Z PPxY mutant peptide was incubated with the pre-washed streptavidin beads in 500 μL of 1× mild buffer for 1 h at 4 °C with rocking. The beads were washed 3X with mild buffer and then incubated with HEK293T cell extracts containing either BAG3-WT, BAG3-ΔN, or BAG3-ΔC. The beads were then washed 3X with 1× mild buffer and suspended in 30 μL of 2× loading buffer with boiling. BAG3 proteins were detected by Western blotting using anti-Myc antibody.

### 2.4. VLP Budding Assays

HEK293T, HAP1-BAG3-WT, or HAP1-BAG3-KO cells were transfected with the LFV-Z expression plasmid using Lipofectamine (Invitrogen, Carlsbad, CA, USA) following the manufacturer’s protocol. After 24 h post-transfection, culture media was centrifuged at 2500 rpm for 10 min to remove cellular debris and layered onto a 20% sucrose cushion in STE buffer (0.01 M Tris-HCl (pH 7.5), 0.01 M NaCl, 0.001 M EDTA (pH 8.0)), and centrifuged at 36,000 rpm for 2 h at 4 °C. The resulting pellet containing LFV-Z VLPs was suspended in STE buffer overnight at 4 °C. Total cell extracts from transfected cells were harvested in RIPA buffer (50 mM Tris (pH 8.0), 150 mM NaCl, 1.0% NP-40, 0.5% deoxycholate, 0.1% SDS) and centrifuged at 13,000 rpm for 5 min to remove cellular debris. The VLPs and cell lysate were fractionated by SDS-PAGE and proteins were detected by Western blotting using the appropriate antisera.

### 2.5. Virus Infection and Titration

HAP1-BAG3-WT or HAP1-BAG3-KO cells were infected with recombinant VSV-M40 [12] at a multiplicity of infection (MOI) of 0.1 for 1 h. The inoculum was removed, and the cells were washed three times with phosphate-buffered saline (PBS). At 8 h post infection, virions were harvested from the media, and titers were determined by standard plaque assays on BHK-21 cells as described previously [17]. Infected cells were lysed in radioimmunoprecipitation assay (RIPA) buffer (50 mM Tris-HCl (pH 8), 150 mM NaCl, 1% NP-40, 0.5% sodium deoxycholate, 0.1% SDS, and protease inhibitors). VSV M protein was detected by SDS-PAGE and Western blotting using anti-VSV-M monoclonal antibody 23H12 kindly provided by D. Lyles (Wake Forest, Winston-Salem, NC, USA).

### 2.6. Confocal Microscopy

HEK293T cells on glass slides were co-transfected with the indicated plasmids for 24 h using Lipofectamine (Invitrogen). Cells were fixed with 4% paraformaldehyde (Affymetrix, Santa Clara, CA, USA) and permeabilized with 0.1% TritonX-100, and then blocked with 5% milk in 1× PBS. Cells were incubated with rabbit polyclonal anti-myc antiserum followed by staining with Alexa488 (green) to detect BAG3 proteins, and with mouse anti-HA antiserum followed by staining with Alexa594 (red) to visualize LFV-Z proteins. Images were acquired on a Leica SP5 inverted confocal microscope with a 100× (NA 1.46) objective lens. The confocal images were subsequently deconvolved with Huygens Essential deconvolution software. Scale bar = 10 μm.

### 2.7. Statistical Analysis

For virus data shown in Figure 4, significance for all statistical tests was determined at *p*-values < 0.05 and is shown as * for *p* < 0.05, ** for *p* < 0.01. VSV titers were log10 transformed before checking normality (via Shapiro Wilks normality test) and assessing equality of variance (via F-test). For samples with unequal variance, Welch’s *t*-test was used to determine statistical significance between log10 VSV titers (*p* = 0.003).

## 3. Results

### 3.1. Identification of Host BAG3 as a Lassa Z PPxY Interactor

**Figure 1:**
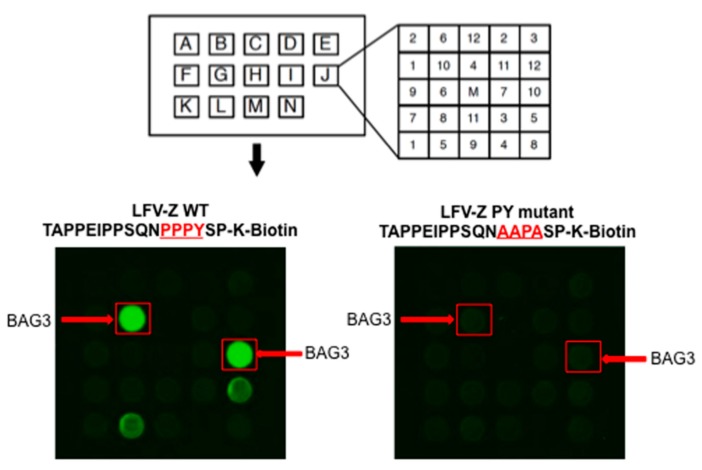
Identification of an interaction between host BAG3 and LFV-Z. Schematic diagram of the GST-WW and GST-SH3 array chip is shown in the top panel. Each lettered square contains one mock (M) and 12 numbered GST-WW or -SH3 domain fusion proteins in duplicate. Fluorescence labeled biotinylated LFV-Z-WT (TAPPEIPPSQNPPPYSP-K-Biotin) and LFV-Z PY mutant (TAPPEIPPSQNAAPASP-K-Biotin) peptides were used to screen the array. A strong interaction between the LFV-Z-WT peptide and the WW-domain of BAG3 was indicated by bright green fluorescent spots shown in the red squares and indicated by the red arrows. No interaction was detected between LFV-Z-PY mutant peptide and BAG3 as shown in the bottom right panel (red squares and arrows).

We used biotinylated peptides containing either the WT (TAPPEIPPSQNPPPYSP-K-Biotin) or a mutated (TAPPEIPPSQNAAPASP-K-Biotin) PPxY motif from the Z protein of Lassa virus to screen an array of 115 GST-WW and 40 GST-SH3 fusion proteins to identify specific host interactors (Figure 1). In addition to other previously known interactors (e.g., Nedd4; data not shown), the LFV-Z-WT peptide interacted robustly with novel host WW-domain bearing proteins including BAG3 (Figure 1, left). Since the LFV-Z-WT peptide did not interact with the majority of GST-WW and GST-SH3 proteins on the array, this suggests that the observed Z-BAG3 interaction was specific (Figure 1). In addition, the Z-BAG3 interaction was dependent on the PPxY motif since the LFV-Z PPxY mutant peptide did not interact with BAG3, nor with any of the arrayed WW or SH3 domains (Figure 1).

**Figure 2:**
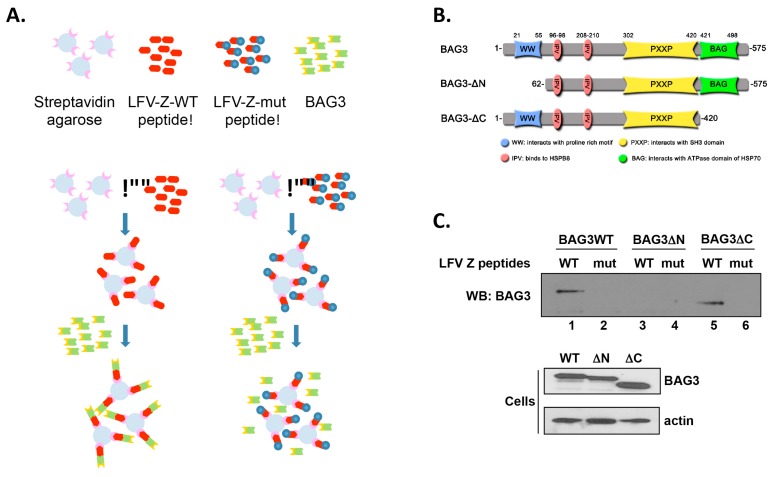
Analysis of viral PPxY-host WW-domain interactions between BAG3 and LFV-Z by peptide pull-down assays. (**A**) Flow chart of the peptide pull-down assay using LFV-Z peptides and cell lysates expressing BAG3-WT; (**B**) Schematic diagram of BAG3-WT, BAG3-ΔN, and BAG3-ΔC mutants with the various domains highlighted in color and amino acid positions indicated; (**C**) Western blot of peptide pull-down assay using streptavidin agarose beads conjugated with either the LFV-Z WT or LFV-Z PY mutant peptide. BAG3 proteins were detected using anti-c-myc antibody (top blot). Expression controls for BAG3 and actin are shown in the bottom blot. These results are from 1 of 2 independent experiments.

To confirm the above Z-BAG3 interaction, we used a peptide pull down approach (Figure 2A) employing the LFV-Z WT and mutant peptides along with full-length BAG3 proteins. Briefly, extracts from HEK293T cells expressing either BAG3-WT, BAG3-ΔN, or BAG3-ΔC (Figure 2B) were incubated with streptavidin agarose beads bound with either the LFV-Z-WT or LFV-Z-mutant peptides. Pulldown proteins (Figure 2C, top) and input protein (Figure 2C, bottom) were detected by Western blotting. Our results indicated that LFV-Z-WT peptide pulled down both BAG3-WT and BAG3-ΔC (Figure 2C, lanes 1 and 5), but not WW-domain deletion mutant BAG3-ΔN (lane 3). The LFV-Z-mutant (mut) peptide did not pull down any of the BAG3 proteins (Figure 2C, lanes 2, 4, and 6). In sum, these data show that the PPxY motif of LFV-Z protein interacted specifically with the WW-domain of novel host interactor BAG3.

### 3.2. Expression of BAG3 Inhibits LFV-Z VLP Egress in a WW-Domain Dependent Manner

**Figure 3:**
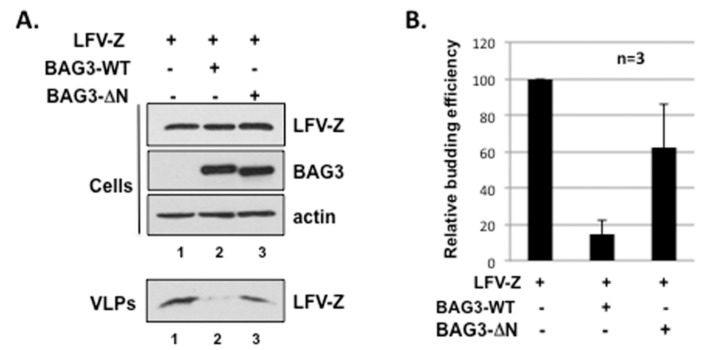
BAG3-WT protein inhibits LFV-Z VLP budding in a PPxY/WW-domain dependent manner. (**A**) Western blot analysis of cell extracts and VLPs from HEK293T cells transfected with LFV-Z alone (lane 1), or LFV-Z + BAG3-WT (lane 2) or BAG3-ΔN mutant (lane 3); (**B**) Relative budding efficiency of LFV-Z VLPs in HEK293T cells transfected as indicated. Error bars represent the standard deviation of the mean from three independent experiments (*n* = 3).

Since we demonstrated above that LFV Z and host BAG3 interact physically, we sought to determine whether this physical interaction would have a biological consequence during budding of LFV VLPs, as we reported previously for budding of filovirus VP40 VLPs. To test this, we used a LFV Z VLP budding assay to ask whether exogenous expression of BAG3-WT or BAG3-ΔN would affect Z VLP egress. Briefly, HEK293T cells were transfected with LFV-Z alone, or in combination with either BAG3-WT or BAG3-ΔN (Figure 3). Cell extracts and supernatants containing Z VLPs were harvested at 24 h post transfection, and proteins were detected and quantified by Western blotting (Figure 3A). In repeated experiments, we observed a consistent decrease in egress of Z VLPs from cells expressing BAG3-WT (Figure 3A, lane 2) compared to those expressing Z alone (lane 1) or BAG3-ΔN (lane 3). Equivalent levels of expression of Z, BAG3, and actin were detected in all cell extract samples (Figure 3A). Results from three independent experiments are shown in the bar graph (Figure 3B). These results indicate that co-expression of LFV Z with exogenous BAG3-WT, but not WW-domain mutant BAG3-ΔN, leads to a decrease in Z VLP egress.

### 3.3. Budding of LFV Z and Ebola VP40 VLPs is Enhanced in BAG3 KO Cells

**Figure 4:**
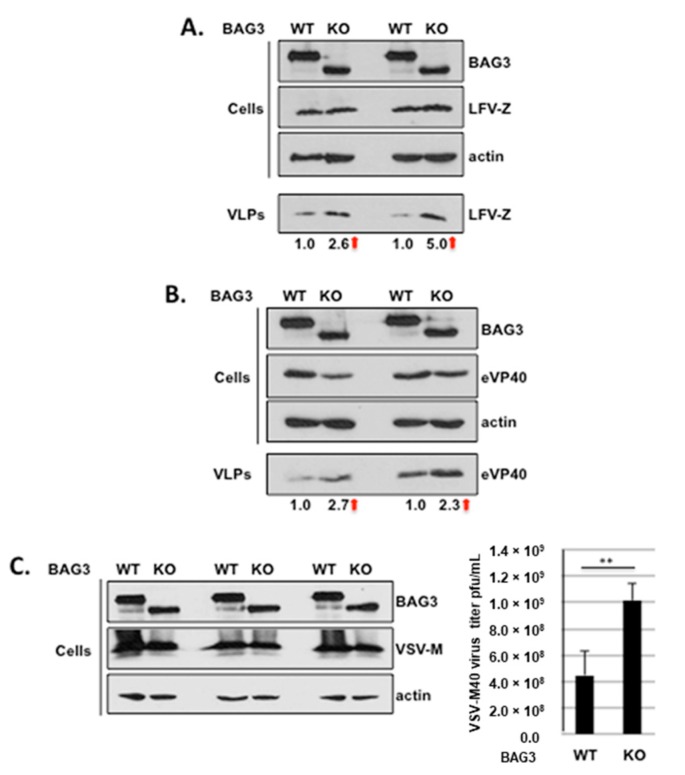
Budding of LFV-Z VLPs, eVP40 VLPs, and VSV-M40 virus from BAG3-WT or BAG3 knockout (KO) cells. (**A**) HAP1-BAG3-WT (WT) or HAP1-BAG3 knockout cells (KO) were transfected with LFV-Z, and the indicated proteins were detected in cell extracts and VLPs by Western blot analysis as shown in two independent experiments; (**B**) HAP1-BAG3-WT (WT) or HAP1-BAG3 knockout cells (KO) were transfected with eVP40, and the indicated proteins were detected in cell extracts and VLPs by Western blot analysis as shown in two independent experiments. The red arrows indicate the fold increase in budding of LFV-Z and eVP40 VLPs in BAG3-KO cells compared to BAG3-WT cells; (**C**) HAP1-BAG3-WT (WT) or HAP1-BAG3 knockout cells (KO) were infected with VSV-M40 recombinant virus, and the indicated proteins were detected in cell extracts by Western blot analysis as shown in three independent experiments. The bar graph depicts the average titers of infectious VSV-M40 virus from three independent experiments. VSV-M40 titers were log10 transformed before checking normality (via Shapiro Wilks normality test) and assessing equality of variance (via F-test). (** *p* = 0.003).

To determine whether endogenous levels of BAG3 could also regulate VLP and virus egress, we made use of parental HAP1 cells expressing WT BAG3, and a HAP1 BAG3 KO cell line that expresses an N-terminally truncated form of BAG3 that is lacking the WW-domain. Briefly, BAG3 WT or KO cells were transfected with WT LFV-Z (Figure 4A) or Ebola VP40 (eVP40) (Figure 4B). Cell extracts and supernatants containing VLPs were harvested at 24 h post-transfection, and the indicated proteins were detected and quantified by Western blotting (Figure 4A,B). Results from two independent Western blots are shown for both LFV-Z and eVP40 VLP budding, in which we consistently observed a modest increase in both Z (Figure 4A) and eVP40 (Figure 4B) VLP egress from BAG3 KO cells compared to that from BAG3 WT cells. This average increase in Z and eVP40 VLP egress of between 2.5–3.0 fold from BAG3 KO cells correlates nicely with our previously published findings for budding of Ebola and Marburg VP40 VLPs from HEK293T cells treated with BAG3-specific siRNAs [19].

As an additional control, we asked whether budding of an infectious VSV recombinant would also be enhanced from BAG3 KO cells compared to BAG3 WT cells (Figure 4C). Briefly, BAG3 WT or KO cells were infected with M40 virus, our VSV recombinant that expresses the PPxY motif and surrounding amino acids from eVP40 in place of the PPxY motif and surrounding amino acids of VSV M [12]. In repeated experiments, VSV-M40 virus titers were found to be significantly higher in BAG3 KO cells compared to those in BAG3 WT cells (Figure 4C). Thus, results from Z VLP, eVP40 VLP, and infectious virus budding assays revealed enhanced levels of egress from cells expressing a truncated, WW-domain deleted form of endogenous BAG3 (KO cells) compared to that from cells expressing WT endogenous BAG3. Taken together, our findings support a role for host BAG3 as a novel LFV Z interactor and negative regulator of Lassa VLP egress.

### 3.4. Confocal Microscopy of Cells Co-Expressing LFV-Z and BAG3

**Figure 5:**
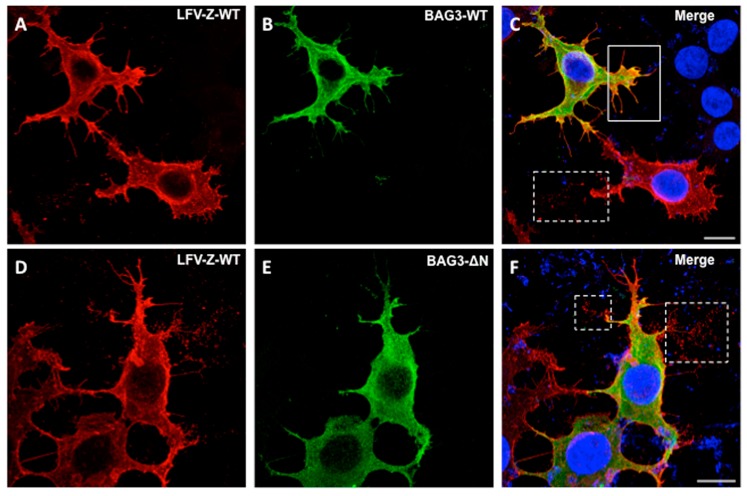
Intracellular localization of LFV-Z and BAG3-WT using confocal microscopy. HEK293T cells were co-transfected with LFV-Z and BAG3-WT (**A**–**C**, top row), or LFV-Z with BAG3-ΔN mutant (**D**–**F**, bottom row). Abundant LFV-Z VLPs (red) were observed to be released from cells co-expressing the BAG3-ΔN mutant (bottom row, merge (**F**), white dotted squares), compared to that released from cells expressing LFV-Z + BAG3-WT (top row, merge (**C**), solid white square). Scale bar = 10 μm.

We next sought to determine whether co-expression of BAG3 would alter the intracellular localization pattern of LFV-Z, leading to a reduced level of VLP egress. To do this, we transfected HEK293T cells with either LFV-Z + BAG3-WT, or LFV-Z + BAG3-ΔN, and then visualized the intracellular localization patterns using indirect immunofluorescence and confocal microscopy (Figure 5). LFV-Z was localized primarily at the plasma membrane (PM) and in PM projections in cells that were also co-expressing BAG3-WT (Figure 5, top row). Interestingly, there appears to be some degree of colocalization of LFV-Z and BAG3-WT at the PM projections; however, we detected little to no release of LFV-Z VLP-like particles from these PM projections in cells co-expressing BAG3-WT, compared to those in cells expressing LFV-Z alone (Figure 5, top row, compare solid white box with dashed white box). In addition, we detected robust release of LFV-Z VLP-like particles from PM projections of cells co-expressing BAG3-ΔN (Figure 5, bottom row, dashed white boxes). These results suggest that BAG3-WT, but not BAG3-ΔN, is playing a role in dampening the release of LFV-Z VLPs perhaps at the site of budding at the PM, and these findings correlate well with results from our VLP budding assays.

## 4. Discussion

The mechanisms by which viral L-domains, such as PPxY, hijack and recruit host proteins to regulate virion egress and spread continue to evolve and remain of great interest, since L-domains are present and functional in a wide array of emerging and deadly RNA viruses. Until recently, all WW-domain bearing host proteins identified as PPxY interactors have exhibited a positive effect on VLP and/or virus budding by enhancing or facilitating release of virus particles. In contrast, we identified BAG3 as the first host WW-domain interactor to negatively regulate egress of Ebola and Marburg VP40 VLPs, as well as infectious virus (VSV-M40) containing the Ebola VP40 PPxY region [19]. In this report, we have extended our findings to show that the WW-domain of BAG3 specifically interacts with the PPxY motif of LFV Z protein, and that BAG3 negatively regulates egress of VLPs produced from LFV Z protein, an arenavirus, in a manner similar to that reported for the filoviruses [19]. Indeed, we detected BAG3 as a LFV Z PPxY interactor using our WW-domain array screen, and validated this interaction by using peptide pulldown assays. Importantly, we found that in addition to the physical interaction between host BAG3 and LFV Z, there was a functional interaction as well as determined by our VLP budding assay, since expression of WT BAG3, but not a WW-domain deletion mutant of BAG3, resulted in a decrease in LFV Z VLP egress. These results lend support to the notion that BAG3 may represent a unique host factor that may function more broadly as part of a novel innate cellular defense mechanism against infections by multiple hemorrhagic fever viruses.

BAG3 is a co-chaperone protein belonging to the BAG family of proteins (BAG1-BAG6), all of which contain a BAG domain that interacts with the ATPase domain of heat shock protein (HSP) 70 [34]. BAG3 is the only WW-domain containing protein in the BAG family, and BAG3 has multiple functions including a role in chaperone-assisted selective autophagy (CASA) to maintain cellular homeostasis under various stressful conditions [35,36,37]. Interestingly, BAG3 and the CASA machinery have been shown to be capable of sensing mechanical tension in muscle cells, and regulate both the synthesis and degradation of filamin; a key actin-crosslinking protein at the plasma membrane that regulates cell adhesion, proliferation, and migration [38,39,40,41,42]. More recently, BAG3, in association with small heat shock protein HSPB8, was shown to play a role in the disassembly of the actin-based contractile ring during cytokinesis [43,44]. Intriguingly, the separation of daughter cells during the final stage of cytokinesis is topologically equivalent to a virus particle separating or pinching off from the cell surface. Indeed, the processes of cytokinesis and virus budding have been linked functionally, as both of these processes require the cellular ESCRT complex and associated factors for their efficient completion [45,46,47,48,49,50,51,52,53]. Thus, it is tempting to speculate that the function of BAG3 and the CASA machinery in regulating quality control of actin and cytoskeletal dynamics under cellular stress or tension, particularly during cytokinesis, may extend to the process of virus budding at the PM, whereby BAG3 and CASA may regulate the actin-based dynamics, stress and membrane tension associated with the pinching off of VLPs and/or viruses [15,54]. Indeed, our confocal microscopic images show that BAG3-WT is co-localized with LFV-Z at the PM and specifically within the PM projections on the cell surface (Figure 5). While results from our confocal microscopy experiments support our speculation, additional experiments would be required to more definitively prove that the negative regulatory function observed for BAG3 on VLP and virus egress is linked to its roles in selective autophagy and quality control of cytoskeletal dynamics.

In sum, we identified host protein BAG3 as a novel WW-domain interactor with LFV Z protein, and as the first host WW-domain bearing protein to negatively regulate egress of LFV Z VLPs. BAG3 has now been shown to negatively regulate egress of VLPs from three different hemorrhagic fever viruses, and thus a better understanding of its role in regulating egress of perhaps an even wider array of viral pathogens is warranted. Indeed, further experiments are required to provide more insight into the potential links between BAG3, selective autophagy, cytoskeletal dynamics and membrane scission events like virus budding. With a more comprehensive understanding of the mechanism by which BAG3 dampens VLP and virus egress, it may be possible to mimic this mechanism and apply it in the design of future antiviral therapies.
